# 2524. Safety and Pharmacokinetics of PA-001, a New Potential COVID-19 Drug That Targets the S2 Subunit of SARS-CoV-2 Spike Protein, in Healthy Subjects

**DOI:** 10.1093/ofid/ofad500.2142

**Published:** 2023-11-27

**Authors:** Haruaki Kurasaki, Masaki Ohuchi, Katsuma Matsui, Masatoshi Matsumoto, Kazutaka Nagatomo, Naoki Kawamura, Shoko Ito, Takuma Yonemura, Takeshi Chiyoda, Asuka Yamamoto, Akihito Shimoi, Keiichi Masuya, Hidetomo Kitamura, Masato Murakami

**Affiliations:** PeptiDream Inc., Kawasaki, Kanagawa, Japan; PeptiDream Inc., Kawasaki, Kanagawa, Japan; PeptiDream Inc., Kawasaki, Kanagawa, Japan; PeptiDream Inc., Kawasaki, Kanagawa, Japan; PeptiDream Inc., Kawasaki, Kanagawa, Japan; PeptiDream Inc., Kawasaki, Kanagawa, Japan; PeptiDream Inc., Kawasaki, Kanagawa, Japan; SOUSEIKAI Sumida Hospital, Tokyo, Tokyo, Japan; SOUSEIKAI Sumida Hospital, Tokyo, Tokyo, Japan; SOUSEIKAI Sumida Hospital, Tokyo, Tokyo, Japan; Ina Research Inc., Ina, Nagano, Japan; PeptiAID Inc., Kawasaki, Kanagawa, Japan; PeptiDream Inc., Kawasaki, Kanagawa, Japan; PeptiDream Inc., Kawasaki, Kanagawa, Japan

## Abstract

**Background:**

Frequent emergence of new variants of the SARS-CoV-2 virus continues to be a concern in treatment of COVID-19 infection, despite the approval of several drugs in recent years. To address this problem, we identified a new macrocyclic peptide, PA-001, which targets the highly conserved S2 subunit of the SARS-CoV-2 spike protein, and confirmed in vivo efficacy in mouse models. Here, we report the clinical safety and pharmacokinetics of PA-001 in healthy subjects (jRCTs031210601).

**Methods:**

Thirty healthy Japanese male volunteers were divided into 5 cohorts (Steps 1 - 5). In each cohort, PA-001 was administered via 1-hour intravenous infusion to 6 subjects at 0.3, 1, 2, 4 or 8 mg, and pharmacokinetics and safety were monitored.

**Results:**

In all cohorts, the plasma concentrations of PA-001 reached C_max_ at 1 hour after administration and decreased with T_1/2_ of 2.30 to 3.39 hours (Figure 1, Table 1). Elimination rate constant (K_el_), CL, V_ss_ and mean residence time (MRT) of PA-001 were 0.216 to 0.309/h, 2,340 to 2,850 mL/h, 9,090 to 11,700 mL and 3.19 to 4.27 h, respectively, and there was no significant difference among each cohort. The 95% confidence interval for the slope using the power model was 0.984 (0.932 to 1.04) for C_max_ and 1.00 (0.941 to 1.07) for AUC_inf_, respectively, confirming the linearity of pharmacokinetic parameters of PA-001 in human plasma.

No serious adverse events were observed in this clinical research. Adverse events occurred in 1 subject from Step 3 (n=6) and in 2 subjects from Step 5 (n=6), while no adverse events were observed in other cohorts. Observed adverse events include: extremity pain (1 subject from Step 3), increase in C-reactive protein levels (1 subject from Step 5) and increase in neutrophile count (1 subject from Step 5). All adverse events were mild and subjects recovered without any additional treatment All adverse events were not causally related to PA-001.

**Figure:**
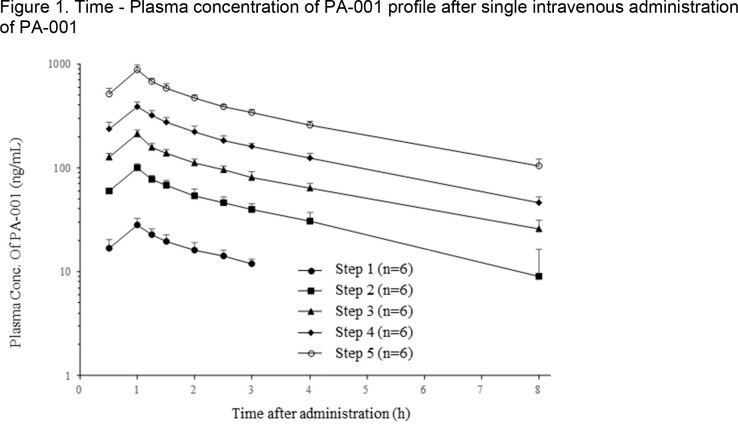

**Figure:**
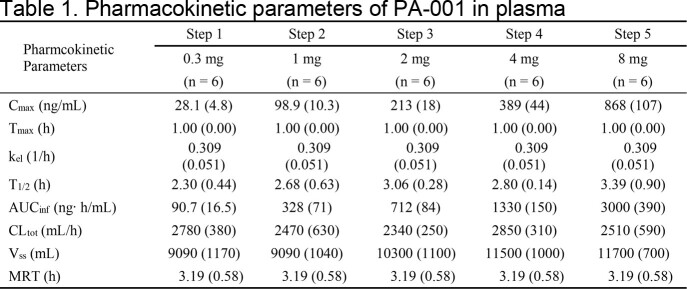

**Conclusion:**

The results showed the safety of PA-001 up to 8 mg when administered as a single intravenous dose over one hour to healthy adult males. Furthermore, PA-001 in plasma was quickly eliminated after administration was completed and there was linearity in the dosage range of 0.3 mg–8 mg. Upon this encouraging data, IND submission for PA-001 is under preparation.

**Disclosures:**

**All Authors**: No reported disclosures

